# Wounding Triggers Wax Biosynthesis in *Arabidopsis* Leaves in an Abscisic Acid–Dependent and Jasmonoyl-Isoleucine-Dependent Manner

**DOI:** 10.1093/pcp/pcad137

**Published:** 2023-10-31

**Authors:** Milena Lewandowska, Krzysztof Zienkiewicz, Agnieszka Zienkiewicz, Amélie Kelly, Stefanie König, Kirstin Feussner, Ljerka Kunst, Ivo Feussner

**Affiliations:** Department for Plant Biochemistry, Albrecht-von-Haller-Institute for Plant Sciences, University of Goettingen, Justus-von-Liebig Weg 11, Goettingen 37077, Germany; Department for Plant Biochemistry, Albrecht-von-Haller-Institute for Plant Sciences, University of Goettingen, Justus-von-Liebig Weg 11, Goettingen 37077, Germany; Service Unit for Metabolomics and Lipidomics, Goettingen Center for Molecular Biosciences (GZMB), University of Goettingen, Justus-von-Liebig Weg 11, Goettingen 37077, Germany; Department for Plant Biochemistry, Albrecht-von-Haller-Institute for Plant Sciences, University of Goettingen, Justus-von-Liebig Weg 11, Goettingen 37077, Germany; Department for Plant Biochemistry, Albrecht-von-Haller-Institute for Plant Sciences, University of Goettingen, Justus-von-Liebig Weg 11, Goettingen 37077, Germany; Department for Plant Biochemistry, Albrecht-von-Haller-Institute for Plant Sciences, University of Goettingen, Justus-von-Liebig Weg 11, Goettingen 37077, Germany; Department for Plant Biochemistry, Albrecht-von-Haller-Institute for Plant Sciences, University of Goettingen, Justus-von-Liebig Weg 11, Goettingen 37077, Germany; Service Unit for Metabolomics and Lipidomics, Goettingen Center for Molecular Biosciences (GZMB), University of Goettingen, Justus-von-Liebig Weg 11, Goettingen 37077, Germany; Department of Botany, University of British Columbia, 6270 University Blvd, Vancouver, British Columbia V6T 1Z4, Canada; Department for Plant Biochemistry, Albrecht-von-Haller-Institute for Plant Sciences, University of Goettingen, Justus-von-Liebig Weg 11, Goettingen 37077, Germany; Service Unit for Metabolomics and Lipidomics, Goettingen Center for Molecular Biosciences (GZMB), University of Goettingen, Justus-von-Liebig Weg 11, Goettingen 37077, Germany; Department for Plant Biochemistry, Goettingen Center for Molecular Biosciences (GZMB), University of Goettingen, Justus-von-Liebig Weg 11, Goettingen 37077, Germany

**Keywords:** Abscisic acid, *Arabidopsis thaliana*, Drought, Jasmonic acid, Wax, Wounding

## Abstract

Wounding caused by insects or abiotic factors such as wind and hail can cause severe stress for plants. Intrigued by the observation that wounding induces expression of genes involved in surface wax synthesis in a jasmonoyl-isoleucine (JA-Ile)-independent manner, the role of wax biosynthesis and respective genes upon wounding was investigated. Wax, a lipid-based barrier, protects plants both from environmental threats and from an uncontrolled loss of water. Its biosynthesis is described to be regulated by abscisic acid (ABA), whereas the main wound signal is the hormone JA-Ile. We show in this study that genes coding for enzymes of surface wax synthesis are induced upon wounding in *Arabidopsis thaliana* leaves in a JA-Ile-independent but an ABA-dependent manner. Furthermore, the ABA-dependent transcription factor MYB96 is a key regulator of wax biosynthesis upon wounding. On the metabolite level, wound-induced wax accumulation is strongly reduced in JA-Ile-deficient plants, but this induction is only slightly decreased in ABA-reduced plants. To further analyze the ABA-dependent wound response, we conducted wounding experiments in high humidity. They show that high humidity prevents the wound-induced wax accumulation in *A. thaliana* leaves. Together the data presented in this study show that wound-induced wax accumulation is JA-Ile-dependent on the metabolite level, but the expression of genes coding for enzymes of wax synthesis is regulated by ABA.

## Introduction

Plants are exposed to many environmental stresses, one of which is mechanical wounding, caused by wind, hail or feeding insects. Wounding leads to cell disruption, production of reactive oxygen species (ROS) and eventually cell death (Orozco-Cardenas and Ryan 1999, Mittler et al. 2011). The membranes and walls of damaged cells lose their integrity, followed by massive metabolic shifts between the different compartments and cells. The neighboring cells seal the wound site to protect the remaining tissue from further damage, pathogen attack or water loss with callus (Ikeuchi et al. 2017), callose (Jacobs et al. 2003), or even lipids such as suberin (Kolattukudy 2001, Domergue et al. 2010). Subsequently, cells surrounding the wound site differentiate and proliferate to regenerate the damaged tissue (Iwase et al. 2011, Sugimoto et al. 2011). Previous reports have shown that wounding also triggers lipid remodeling and accumulation of neutral lipids, such as triacylglycerols (Vu et al. 2014, 2015, Lewandowska et al. 2023). Transcriptomic studies indicated that this might also be true for another class of neutral lipids, namely waxes.

The barrier protecting the plant from desiccation, UV radiation and organ fusion is the cuticle. It covers all aerial plant organs, except for the stems of woody plants, and it is composed of cutin and wax (Cook and Graham 1998). Cutin is a fatty acid- and glycerol-based polyester forming an insoluble polymer that functions as a scaffold (Fich et al. 2016). However, wax is a combination of aliphatic compounds surrounding and covering cutin and therefore the plant surface. In *Arabidopsis thaliana*, waxes are primarily composed of very-long-chain fatty acids (VLCFAs) and their derivatives including alkanes, aldehydes, alcohols, ketones and wax esters, which typically range from 24 to 34 carbons in length. Wax biosynthesis takes place in the epidermal cells. It starts with the successive elongation of fatty acyl-CoAs, which are then fed into two major routes: the alcohol-forming pathway leading to the formation of wax esters and the alkane-forming pathway resulting in alkanes (Samuels et al. 2008). The different wax components are subsequently transported through the cell wall to the outer layer. Sometimes, there is an additional layer of crystal-like structures or non-crystalloid films known as epicuticular wax, which provides a further measure to prevent infestation with bacteria, fungi or even insects (Lewandowska et al. 2020).

In angiosperms, the wound response as such is known to be mainly orchestrated by the active form of jasmonic acid (JA), namely jasmonoyl-isoleucine (JA-Ile), which regulates the expression of defense genes (Farmer et al. 2014, Howe et al. 2018, Wasternack and Feussner 2018). The amount of JA-Ile increases rapidly after wounding (Koo and Howe 2009). In *A. thaliana*, JA-Ile binds to a co-receptor complex composed of the JASMONATE ZIM domain (JAZ) and an E3 ubiquitin ligase complex CORONATINE INSENSITIVE1 (COI1). This interaction triggers the degradation of JAZ and the de-repression of MYC transcription factors that initiate JA-dependent expression of defense-related genes (Howe et al. 2018). Mutants deficient in the precursors of JA, such as α-linolenic acid (18:3) or roughanic acid (16:3), cannot mount a sufficient defense against insects (McConn et al. 1997), and mutants of *COI1* are more susceptible to necrotrophic fungi (Thomma et al. 1998). Moreover, plants treated with JA or methyl-jasmonate (MeJA) exhibit a greater defense response against herbivores and necrotrophs (Thomma et al. 1998, Kessler and Baldwin 2001). Another mutant deficient in JA biosynthesis due to disrupted *ALLENE OXIDE SYNTHASE* (*AOS*), designated *dde2-2*, accumulates more callus around the wound site than wild-type (WT) plants (Ikeuchi et al. 2017).

A second plant hormone synthesized in response to wounding is abscisic acid (ABA) (Bostock and Stermer 1989, Pena-Cortes et al. 1995), which also plays a major role in drought or osmotic stress–induced signaling (Cutler et al. 2010). In plants, ABA is derived from violaxanthin, with ABSCISIC ALDEHYDE OXIDASE3 (AAO3) (Nambara and Marion-Poll 2005) committing the last step of its synthesis. When ABA levels increase, the ABA receptor complex PYRABACTIN RESISTANCE1 (PYR)/PYR1-like (PYL)/REGULATORY COMPONENT OF ABA RECEPTOR1 (RCAR) binds both ABA and the 2C-TYPE PROTEIN PHOSPHATASE (PP2C) repressor. This leads to the activation of the SNF1-RELATED PROTEIN KINASE2 (SnRK2) and consequently to the phosphorylation and activation of many downstream transcription factors (Raghavendra et al. 2010, Nakashima and Yamaguchi-Shinozaki 2013, Cui et al. 2016). One of such an ABA-dependent and stress-activated transcription factor is MYB96, which is known for its involvement in pathogen defense (Seo and Park 2010) and drought response (Seo et al. 2009, Lee et al. 2016). Additionally, together with MYB94, it also regulates the first step of wax biosynthesis precursors (Seo et al. 2011, Lee et al. 2016), by controlling the expression of *ECERIFERUM2* and *ECERIFERUM6* (*CER2* and *CER6*). A similar situation was found for MYB94 in corn (Castorina et al. 2020). *CER6* encodes the β-KETOACYL-CoA SYNTHASE6, which catalyzes the first reaction of elongation of VLCFA and is the rate-limiting enzyme for wax biosynthesis (Millar and Kunst 1997, Fiebig et al. 2000, Hooker et al. 2002). CER10 is an enoyl-CoA reductase and is the last enzyme of the fatty acid elongation pathway (Zheng et al. 2005, Bach et al. 2008). CER2 is a protein of still unknown function, yet required for the extension of VLCFAs to lengths >28 carbons (Haslam et al. 2012). Furthermore, MYB94 and MYB96 regulate the expression of selected genes from both the alkane-forming pathway, *ECERIFERUM1 (CER1) and ECERIFERUM 3* (*CER3*), and the alcohol-forming pathway, *WAX SYNTHASE/ACYL-CoA:DIACYLGLYCEROL ACYLTRANSFERASE1* (*WSD1*) (Lee et al. 2016). Likewise, the expression of two other genes encoding wax export components, namely *LIPID TRANSFER PROTEIN G2* (*LTPG2)* and *ATP BINDING CASSETTE G11* (*ABCG11)*, are also under the control of MYB96 (Seo et al. 2011).

In a previous study, datasets from both transcriptome and metabolome analyses performed on wounded WT and JA-deficient *dde2-2* plants were combined to enhance the identification of metabolites and corresponding pathways that play a role in the JA-Ile-dependent wound response of *A. thaliana* (Kaever et al. 2015). This approach suggested that surface wax biosynthesis is a substantial part of the local wound response and that its regulation might be indeed partly JA-Ile-independent, as shown for transcripts for *CER2* and *CER10* and the two already known transcription factors *MYB94* and *MYB96* (**Supplementary Table S1**). Indeed, earlier work revealed that wax formation is regulated by the MYB96 transcription factor (Seo et al. 2011, Lee et al. 2018), which in turn is known to be induced by the plant drought hormone ABA (Seo et al. 2009). Therefore, we included a knockout mutant of MYB96 *(myb96-1)* and a mutant with reduced ABA content (*aao3-4*) in our investigations to determine if ABA signaling plays a role in wax biosynthesis upon wounding. Focusing on the local wound response of whole rosette leaves, we show that on the metabolite level, wound-induced wax accumulation is dependent on JA-Ile biosynthesis. However, the expression of genes coding for enzymes of wax synthesis is regulated by ABA biosynthesis via MYB96.

## Results

### Wounding leads to a strong JA-Ile-independent yet ABA-dependent accumulation of transcripts related to wax biosynthesis

JA-Ile orchestrates the plant response to wounding (Howe et al. 2018), while ABA regulates drought stress–dependent reactions (Nakashima and Yamaguchi-Shinozaki 2013). In order to distinguish JA-Ile as well as ABA-dependent and ABA-independent responses upon wounding, WT plants, the JA-deficient *dde2-2* mutant and a mutant with reduced ABA content and synthesis (*aao3-4*) were used for transcript, wax and phytohormone analyses. In addition, loss-of-function mutants for the transcription factor MYB96 (*myb96-1*) *and* MYB94 (*myb94-1*) were included because of their known involvement in the regulation of wax biosynthesis and ABA-dependent defense responses.

Gene expression analysis was performed on samples harvested at time points of 0, 0.5, 2 and 5 h past wounding (hpw). They were chosen to cover processes of the early wound response, in analogy to the experiments performed before (Kaever et al. 2015).

**Fig. 1:**
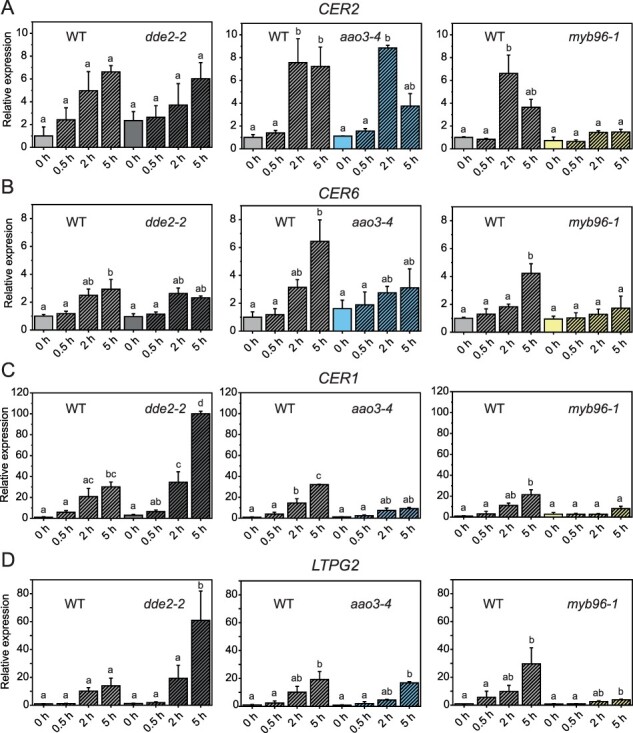
Expression of genes involved in wax biosynthesis is induced upon wounding. Transcript of chosen genes was analyzed by qRT-PCR in leaves of WT, *dde2-2*, *aao3-4* and *myb96-1* before (0 h), 0.5, 2 and 5 h post wounding. Relative expression of the following transcripts is shown: (A,B) *CER2* and *CER6* involved in fatty acid elongation; (C) *CER1* involved in aldehyde/alkane formation; (D) *LTPG2* coding a wax transporter. Values represent means (±SE) of qRT-PCR analyses of plants of three independent experiments. Letters indicate statistical significance determined by ANOVA and Tukey’s post-hoc test (*P *< 0.05). Data of this figure are available in **Supplementary Table S1**.

Applying quantitative real-time PCR (qRT-PCR) analysis to the key genes of wax biosynthesis, we found that all are upregulated in response to wounding in a JA-Ile-independent manner (**Fig. 1**, **Supplementary Fig. S1**). Genes encoding enzymes of the initial steps of wax biosynthesis, *CER2, CER6* and *CER10*, are induced in WT plants within 2 and 5 hpw c. 4- to 6-fold. Interestingly, genes encoding enzymes involved in the later steps of wax biosynthesis and wax transport to the cuticle, such as *CER1* and *LTPG2*, showed a much higher expression in *dde2-2* at 5 hpw than WT. For these genes, 100- and 60-fold increase, respectively, of transcript accumulation was detected in *dde2-2* leaves compared to a 30-fold increase for *CER1* and a 20-fold increase for *LTPG2* measured in WT leaves (**Fig. 1C, D**). The wax biosynthesis regulator *MYB96* was also more strongly upregulated in *dde2-2* plants in comparison to WT (**Supplementary Fig. S2**). qRT-PCR analysis of ABA-deficient wounded *aao3-4* leaves showed a significant induction of *CER2, WSD1* and *LTPG2* expression (**Fig. 1A, D** and **Supplementary Fig. S1B**), whereas only a slight induction was observed for *CER6*, *CER10*, *CER1, CER3* and *ABCG11* (**Fig. 1B, C** and **Supplementary Fig. S1A, C, D**). None of the tested genes were significantly upregulated in the *myb96-1* mutant. Also, *myb94-1* plants were analyzed, but no change in comparison to WT could be shown (**Supplementary Fig. S3**).

### JA-Ile is essential for cuticular wax accumulation after wounding

**Fig. 2:**
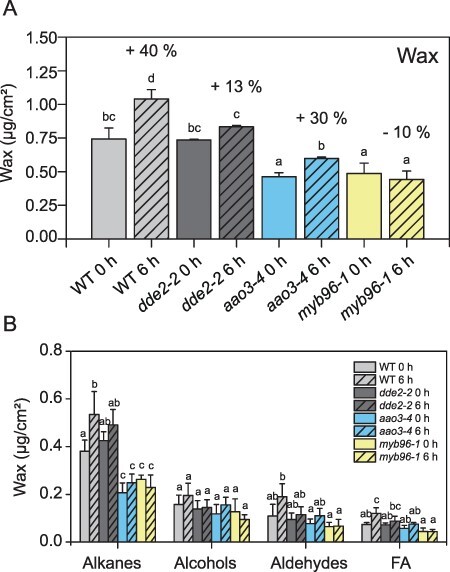
Wax accumulates upon wounding. (A) Total wax load in *Arabidopsis* leaves before (0 h) and 6 h post wounding. (B) Wax compound (wax, alkanes, aldehydes and fatty acids) amounts before and 6 h after wounding. Each compound group was statistically analyzed separately. Values are means (±SD) of GC-FID analyses of plants from three independent experiments. Letters indicate statistical significance determined by ANOVA and Tukey’s post-hoc test (*P *< 0.05). Data of this figure are available in **Supplementary Table S4**.

Based on the gene expression profiles (**Fig. 1**, **Supplementary Fig. S1**), which showed the highest expression rate at 2 and 5 hpw, we analyzed the total wax content in leaf cuticles at 0 and 6 hpw to allow for an appropriate accumulation of the respective metabolites. Since the goal was to analyze wax production without further wounding the leaf, we extracted waxes by dipping whole leaves in chloroform without further dissecting them. Therefore, it was not possible to analyze wax production at a higher spatial resolution closer to the wounding site. Measurement by GC-FID revealed that in WT, c. 40% more wax was present at 6 hpw in comparison to the non-wounded control leaves (**Fig. 2A**). This increase was due to significant increases in both alkane and fatty acid contents (**Fig. 2B**). Surprisingly, although WT and *dde2-2* leaves had similar wax loads before wounding, *dde2-2* leaves did not exhibit a significantly increased wax deposition in response to wounding, unlike WT leaves. *aao3-4* leaves instead had lower wax load in control samples than WT leaves (0.74 µg/g fresh weight (FW) vs. 0.46 µg/g FW), but wax load increased by 30% after wounding (**Fig. 2A**).

It was previously shown that *myb96-1* accumulates less wax on leaves and stems than WT although the expression of genes involved in wax biosynthesis was not changed in *myb96-1* plants under normal growth conditions (Seo et al. 2011). Our data confirmed these findings and showed that wax load of *myb96-1* was reduced by 40% relative to WT (**Fig. 2A**), with the greatest decrease detected for alkanes (**Fig. 2B**). Wounding did not change wax load significantly in those plants (**Fig. 2A**). Untreated *myb94-1* plants showed a similar reduction as *myb96-1* mutants in relation to WT plants; however, after wounding, the wax load increased similar to WT (**Supplementary Fig. S3C**).

**Fig. 3:**
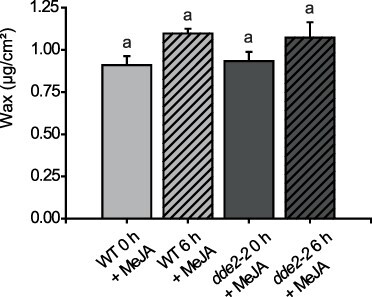
Wax analysis of plants sprayed with 5 mM MeJA before (0 h) and after wounding (6 h) in WT and the JA-deficient mutant *dde2-2*. Values represent means (±SD) of GC-FID analyses of plants harvested from one single-wounding experiment. A second experiment with similar results was conducted. Letters indicate statistical significance determined by ANOVA and Tukey’s post-hoc test (*P *< 0,05). Data from both experiments are available in **Supplementary Table S5**.

Currently, there is no published evidence of a connection between JA-Ile and leaf cuticular wax content. To examine the effect of JA-Ile on wax accumulation in response to wounding, we attempted to recover the WT response to wounding in *dde2-2* plants by external application of MeJA. MeJA was sprayed on WT and *dde2-2* mutant plants 24 h before the wounding experiment. Interestingly though the wax content may have slightly increased on the non-wounded WT plants in comparison to the plants growing without MeJA treatment, the treatment did not influence the wound-induced wax content in WT (**Figs. 2**, **3**). For the JA-Ile-deficient mutant *dde2-2*, wax accumulation was similar to that of WT plants after MeJA application and wounding (**Fig. 3**).

### Plants deficient in JA-Ile biosynthesis accumulate salicylic acid and callose upon wounding

**Fig. 4:**
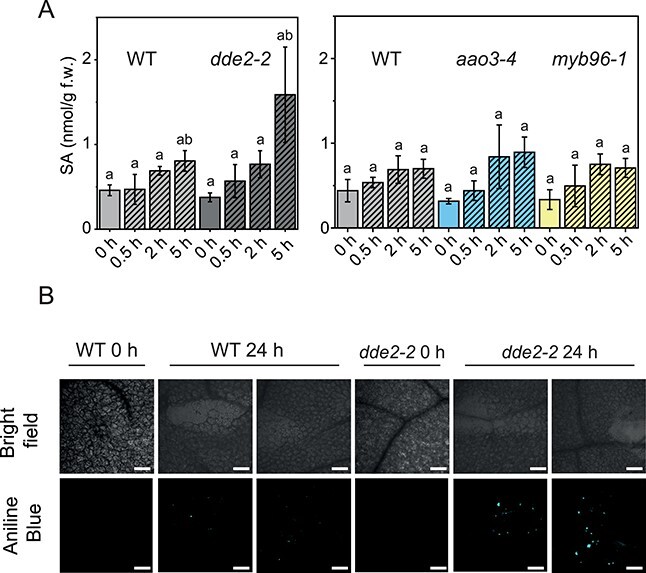
Analysis of SA content and callose deposition. (A) SA content in WT, d*de2-2, aao3-4* and *myb96-1*. Values represent means (±SD) of LC-MS/MS analysis of plants harvested from three independent wounding experiments. Alphabetical letters indicate statistical significance determined by ANOVA and Tukey’s post-hoc test (*P *< 0.05). Data of this figure are available in **Supplementary Table S6**. (B) Analysis of callose formation in WT and *dde2-2* before and 24 h after wounding. Analysis was performed by confocal microscopy, bars: 300 µm.

It is well established that JA-Ile and salicylic acid (SA) can work antagonistically (Robert-Seilaniantz et al. 2011). SA induces callose deposition in *A. thaliana* (Wang et al. 2013), which is also formed after wounding (Jacobs et al. 2003). To further investigate why wounding of JA-Ile-deficient *dde2-2* leaves did not result in increased wax deposition, we quantified the amount of SA and analyzed callose deposition in mutant leaves and WT before and after wounding. WT plants as well as *aao3-4* and *myb96-1* plants did not show a prominent increase in SA up to 5 hpw; however, in the *dde2-2* mutant, SA accumulation was substantially higher although the increase was not statistically significant at 5 hpw (**Fig. 4A**). Moreover, a higher number of callose plugs were observed in the wounded *dde2-2* plants at 24 hpw in comparison to the wounded WT plants (**Fig. 4B**).

### High humidity prevents wound-induced wax accumulation in *A. thaliana* leaves

**Fig. 5:**
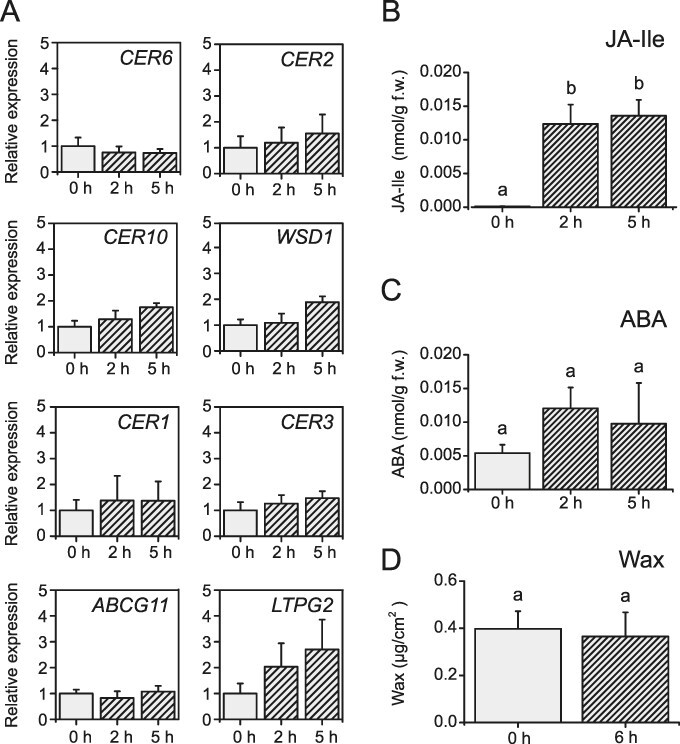
High humidity prevents the accumulation of ABA and wax in response to wounding in *Arabidopsis* leaves. (A) Expression of genes involved in wax biosynthesis in non-wounded (0 h) and wounded (2 and 5 h) WT plants determined by qRT-PCR. None of the tested genes were significantly upregulated. Quantification of (B) JA-Ile and (C) ABA in non-wounded (0 h) and wounded (2 and 5 h) WT plants by LC-MS/MS. (D) Total surface wax content in *Arabidopsis* leaves before (0 h) and 6 h post wounding. Values represent means (±SD) of analyses of plants from three independent experiments. Letters indicate statistical significance determined by ANOVA and Tukey’s post-hoc test (*P *< 0.05). There is no statistical difference for the data in (A), and hence, letters are not shown for this part. Data of this figure are available in **Supplementary Table S7**.

Because ABA is not only involved in wound-induced wax accumulation (**Fig. 2A**) but also a key drought stress hormone, it is necessary to determine if the detected increase in wax accumulation is a result of wounding or drought stress. *Arabidopsis thaliana* WT plants were therefore grown and wounded at high humidity (∼96%), and the expression of genes involved in wax formation before and after wounding was analyzed. None of the wax biosynthetic genes were upregulated significantly after wounding in high humidity (**Fig. 5A**), even though plants responded to the stress treatment as indicated by elevated amounts of JA-Ile at 0.5 and 2 hpw (**Fig. 5B**). Interestingly, the amount of JA-Ile was c. 10-fold lower in wounded plants grown in high humidity than in wounded plants grown under normal humidity conditions (**Fig. 5B**, **Supplementary Fig. S4B**). In contrast to JA-Ile, there was no significant increase in ABA amount after wounding (**Fig. 5C**). Upon wounding under normal humidity conditions, ABA amount increased 4-fold up to 2 hpw (**Supplementary Fig. S4A**), whereas under high humidity conditions, this increase was up to ∼2.5-fold 2 hpw, but this difference is not significant. We also assessed the wax load on *A. thaliana* leaves grown and wounded under high humidity. Whereas a 40% increase in wax load was observed in wounded leaves in comparison to non-wounded plants under normal humidity conditions (**Fig. 2A**), wax content did not change after wounding at high humidity (**Fig. 5D**).

### Wax biosynthesis is not induced in *A. thaliana* inflorescence after wounding

**Fig. 6:**
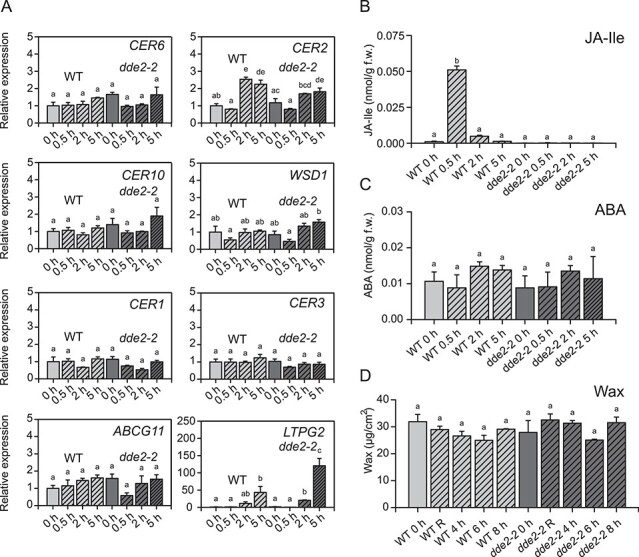
Wounding of *Arabidopsis* inflorescence stems does not trigger wax biosynthesis. (A) Expression of genes involved in wax biosynthesis before (0 h) and after wounding (2 and 5 h) in WT and the JA-Ile-deficient mutant *dde2-2*. The transcript was analyzed by qRT-PCR. Quantification of (B) JA-Ile and( C) ABA in non-wounded (0 h) and wounded (2 and 5 h) inflorescence stems of WT and *dde2-2* by LC-MS/MS. (D) Wax load of non-wounded (0 h), wounded and immediately extracted (R) inflorescence stems as well as stems harvested 4, 6 or 8 h post wounding were analyzed by GC-FID. Values represent means (±SD) of analyses from three independent experiments. Letters indicate statistical significance determined by ANOVA and Tukey’s post-hoc test (*P *< 0.05). Data of this figure are available in **Supplementary Table S8**.

Inflorescence stems of *A. thaliana* are covered by 40 times more cuticular wax than leaves (**Figs. 2A**, **6D**). We investigated whether wax biosynthesis in inflorescence stems can also be stimulated by wounding. qRT-PCR analyses performed on wounded and non-wounded inflorescence stems of WT and *dde2-2*-mutant plants did not reveal a significant induction of genes involved in wax biosynthesis after wounding (**Fig. 6A**), with the exception of *CER2* and *LTPG2*, which showed higher transcript accumulation. As expected, the JA-Ile-dependent wound response was initiated in the inflorescence stems immediately after wounding, indicated by a rapid increase in JA-Ile levels in WT, but not in the *dde2-2* mutant (**Fig. 6B**). Surprisingly, no induction of ABA was observed in either plant line (**Fig. 6C**) although ABA can be produced and transported in inflorescence stems (Hartung et al. 2002, Koiwai et al. 2004). The wax amount was measured at 4, 6 and 8 hpw in inflorescence stems (**Fig. 6D**), with an additional sample (R) harvested just after wounding. This sample was analyzed to exclude potential mechanical abrasion of wax as a result of the wounding procedure. Changes in wax content after wounding were not detected in WT or *dde2-2* plants.

## Discussion

Previous work demonstrated that plants can use different materials to seal wound sites, including callose, callus and suberin (Kolattukudy 2001, Jacobs et al. 2003, Domergue et al. 2010, Ikeuchi et al. 2017). In this study, we focused on the wound response in *Arabidopsis* leaves since this large set of mutants that we aimed to analyze was only available for this model plant. We show that wounding enhances the production of leaf surface wax and that this process is dependent on the wound response hormones JA-Ile on the metabolite level and the drought stress hormone ABA on the transcript level. In addition, the transcriptional regulation by ABA is mainly regulated via MYB96.

### Wax biosynthesis upon wounding is ABA-dependent at the transcript level, and MYB96 is a major regulator of this process

To determine if cuticular wax biosynthesis and deposition are affected by wounding, we analyzed the expression of wax-related genes and determined wax load on wounded WT, *dde2-2, aao3-4* and *myb96-1* leaves (**Figs. 1**, **2A**). Wounding enhanced the expression of all tested genes, and increased gene expression was independent of JA-Ile but dependent on ABA. Furthermore, some of the wax biosynthetic genes, including *CER1, LTPG2* and *MYB96*, exhibited even substantially higher expression in the JA-Ile-deficient *dde2-2* mutant than in WT upon wounding. To date, ABA was the only plant hormone known to affect wax biosynthesis. Increased ABA levels activate transcription factors such as MYB94 or MYB96 (Broun et al. 2004, Lee and Suh 2013, Castorina et al. 2020). It was previously shown that the ABA amount increases after wounding, which we confirmed in our studies (**Supplementary Fig. S4**) (L’Haridon et al. 2011). Our analysis of the ABA-reduced mutant *aao3-4* revealed that in these plants ABA levels also increase c. 2-fold in response to wounding, but ABA accumulation is less pronounced than in WT plants. Since *AAO3* is not the only abscisic aldehyde oxidase in *A. thaliana*, it is not surprising that the *aao3* mutant has a subtle phenotype (Seo et al. 2000). Gene expression analyses of *aao3-4* plants showed that only some of the genes involved in wax biosynthesis are upregulated after wounding (**Fig. 1**). Similar results were obtained for ABA-signaling mutants in *A. thaliana*, e.g. mutants deficient in SNF1-Related Protein Kinase 2 (Cui et al. 2016), and the ABA-reduced lines of tomato where decreased expression of *CER3, ABCG11* and *CER6* genes also resulted in lower wax accumulation (Martin et al. 2017). Wax analysis of *A. thaliana aao3-4* plants demonstrated that the wax load was also reduced in comparison to WT in non-wounded and wounded plants (**Fig. 2A**). Thus, we concluded that elevated ABA levels contribute to induction of wax biosynthesis in response to wounding. Because MYB94 and MYB96 transcription factors are known to work additively in activating wax biosynthesis in an ABA-dependent manner (Lee et al. 2016), we investigated gene expression in *myb96* and *myb94* mutants. Analysis of *myb96-1* showed in contrast to WT plants that neither the transcripts involved in wax biosynthesis nor the wax amounts were enriched significantly after wounding, an indication that MYB96 is a key regulator of wax biosynthesis in response to wounding. In contrast to *myb96, myb94* mutant displayed a similar wound response as WT plants (**Supplementary Fig. S3**), suggesting that MYB94 transcription factor is not directly involved in wax deposition after wounding.

### JA-Ile might regulate wax biosynthesis upon wounding post-translationally

Surprisingly, despite the induction of key wax genes, wounding did not lead to an increase in wax content in the *dde2-2* mutant as observed in WT plants (**Figs. 1**, **2**), but wax accumulation could be restored to WT levels in *dde2-2* by external application of MeJA (**Fig. 3**). Thus, our work uncovered a previously unknown link between JA-Ile and wax biosynthesis in the wound response suggesting for a post-translational JA-Ile-dependent regulation of enzymes or transporters being involved in wax accumulation upon wounding. However, knowledge of the post-translational regulation of wax biosynthesis is still scarce, and further studies are needed to shed light on this phenomenon. In addition, it was shown that callose deposition after wounding in leaves was more pronounced in *dde2-2* (**Fig. 4B**). The function of callose in the wound response is sealing the injured area to protect the damaged plant from pathogen penetration (Jacobs et al. 2003). This process is known to be regulated by SA (Wang et al. 2013). In line with this, we observed in *dde2-2* plants, which lack JA-Ile, a 2-fold increase of SA in comparison to WT plants at 5 hpw (**Fig. 4A**). Perhaps, callose serves a similar function in sealing the wound site and might substitute surface wax.

### Wax biosynthesis after wounding is drought stress dependent

Since MYB96 is a transcription factor that controls wax biosynthesis in response to drought, and ABA is a main drought-related signaling hormone, it was necessary to dissect wound and drought stress responses. Wounding of *A. thaliana* leaves in high humidity showed that neither enhanced expression of genes governing wax biosynthesis nor wax accumulation took place upon wounding (**Fig. 5**). In addition, JA-Ile amount was 10-fold lower upon wounding in high humidity conditions than upon wounding in normal humidity (**Supplementary Fig. S4** and **Fig. 5B**). Furthermore, under these conditions, the level of ABA after wounding remained unchanged. These data are consistent with previous reports that plants wounded in high humidity do not accumulate ABA upon wounding. These plants also exhibit higher cuticular permeability than control plants wounded under normal humidity conditions (L’Haridon et al. 2011). This suggests that in high humidity, plants do not seal wound sites as efficiently as they do under normal conditions since the risk of water loss is diminished. Previous studies established that wax accumulates during drought stress in WT *A. thaliana* leaves as well as in *myb96-1* plants, however in reduced amounts (Seo et al. 2011). Furthermore, it was recently shown that wax esters, which are relatively minor components of *A. thaliana* cuticular wax, accumulate in response to drought stress in leaves and stems of *Arabidopsis* (Patwari et al. 2019). However, we were not able to detect any wax esters in leaves under our conditions most likely because their levels were below the detection limit of our analysis. Genes associated with drought stress–related ABA signaling, *RESPONSIVE TO DESSICATION 29A* (*RD29A)* and *ABSCISIC ACID INSENSITIVE* 1 (*ABI1)*, were strongly induced by wounding (**Supplementary Fig. S5**). Thus, wound-induced wax biosynthesis shares similarities with drought-induced wax formation. However, these processes differ with respect to the involvement of MYB94, which is not involved in the wound-induced response (**Supplementary Fig. S3**). It should be also taken into consideration that upon wounding in high humidity JA-Ile signaling might be impaired since less JA-Ile was produced under those conditions (**Supplementary Fig. S4** and **Fig. 5B**), and as we have shown before, induced amounts of JA-Ile and ABA are necessary for wound-induced wax accumulation (**Fig. 2A**). Taken together, our results provide strong evidence that wax accumulation upon wounding is part of drought stress response in leaves.

*Arabidopsis thaliana* inflorescence stems bear 40-fold more wax than leaves, but they responded similarly to leaves wounded under high humidity. Neither organ exhibited an enhanced expression of wax biosynthetic genes, greater wax deposition, or ABA accumulation, even though elevated levels of JA-Ile were detected (**Fig. 6**). However, wax biosynthesis might be already suppressed in inflorescence stems since they contain much more wax than *A. thaliana* leaves from the beginning.

In conclusion, we demonstrated that surface wax accumulates upon wounding in *A. thaliana* leaves and that this process is dependent on elevated amounts of JA-Ile and ABA and MYB96 as a key regulator. Furthermore, it is possible that this accumulation is dependent on drought stress sensed by plants after wounding. We propose a model in which wounding induces an increase in JA-Ile and ABA content resulting in the activation of MYB96 and enhanced wax biosynthesis, most likely to seal the site of wounding, due to its hydrophobic properties; however, future studies are needed to fully confirm its function.

## Materials and Methods

### Plant materials and growth conditions

The following *A. thaliana* lines were used in this study: WT Columbia-0 ecotype, *dde2-2* (von Malek et al. 2002), *aao3-4* (kindly provided by Prof. Dr Christiane Gatz, University of Goettingen), *myb96-1* (Seo et al. 2009) and *myb94-1* (Lee and Suh 2014) (both kindly provided by Prof. Dr Mi Chung Suh, Chonnam National University). All plants were grown in growth chambers under the following conditions, unless specified otherwise: white light illumination (130–150 µmol m^−2^ s^−1^) under short-day conditions (8 h light: 16 h dark) at 22°C during the day and at 18°C at night, with c. 60% humidity. For experiments with inflorescence stems, plants were grown under long-day conditions (16 h light: 8 h dark). For high humidity experiments, plants were grown in trays tightly sealed with plastic lids and covered with plastic foil. Five days before wounding, plants were transferred to a chamber with c. 90% humidity at night and 96% humidity during the day. Plants were grown on soil supplemented 4:1 with vermiculite.

### Stress treatment

Rosette leaves of 6- to 7-week-old plants were mechanically wounded using forceps (Stenzel et al. 2003). Inflorescence stems of 4- to 5-week-old plants were wounded every 0.5 cm from the bottom up to the first siliques. For gene expression profiling and phytohormone measurements, wounded rosettes or inflorescence stems were harvested and immediately frozen in liquid N_2_. For each experiment, six to 10 plants were pooled at each time point. For expression and phytohormone analysis, plants were harvested at 0, 0.5, 2 and 5 hpw, with the exception of high humidity experiments where the time point of 0.5 hpw was skipped. For MeJA treatment, plants were sprayed with 5 mM MeJA in 0.1% aqueous Tween20 solution 24 h before wounding.

### Determination of phytohormones by ultra high pressure liquid chromatography (UPLC)-nano electrospray ionization (ESI)-tandem mass spectrometry (MS/MS)

Analysis was performed as described (Kusch et al. 2019). Plant material (100 mg) was extracted with 0.75 ml methanol containing 10 ng of each of the following deuterated internal standards: D_4_-salicylic acid (D_4_-SA), D_6_-ABA, D_5_-JA (C/D/N Isotopes Inc., Pointe-Claire, Canada) and D_4_-JA-Leu (kindly provided by Otto Miersch, Halle/Saale, Germany). Mass transitions are listed in **Supplementary Table S2**.

### Analysis of transcript levels

qRT-PCR analysis was used to investigate gene expression levels. Total RNA was extracted using TRI-reagent (Sigma-Aldrich, armstadt, Germany) according to the manufacturer’s protocol. RNA quantification was carried out with a NanoDrop 2000 spectrophotometer (Thermo Fisher Scientific, Waltham, MA). One microgram of RNA was treated with DNase I (Thermo Fisher Scientific), and cDNA was synthesized using Revert Aid H Minus Reverse Transcriptase (Thermo Fisher Scientific). qRT-PCR was performed using Takyon No ROX SYBR Mastermix blue dTTP (Kaneka Eurogentec, Seraing, Belgium) in a 20-µl reaction volume. The *ACTIN8* gene was used as a reference. All primers used in this experiment are listed in **Supplementary Table S3**. Each reaction was performed with material from plants harvested in three independent experiments in an iQ5 real-time detection system (Bio-Rad, Feldkirchen, Germany).

### Wax extraction and analysis

Wax was extracted from leaves of 6- to 8-week-old plants and stems of 4- to 5-week-old plants. Stems or leaves were immersed in chloroform containing tetracosane (Sigma-Aldrich) as an internal standard. Samples were then dried under an N_2_ stream and re-dissolved in 10 µl of *N, O*-bis(trimethylsilyl)trifluoroacetamide (Sigma-Aldrich) and 10 µl of pyridine (Sigma-Aldrich). Derivatization was performed at 80°C for 1 h. Afterward, samples were dried under an N_2_ stream and re-dissolved in 10 µl of chloroform. Wax was quantified by gas chromatography with a flame ionization detector (Agilent GC 6890, Agilent Technologies, Waldbronn, Germany) coupled with a 30-m HP-1 column using helium as a carrier gas. Two microliters of each sample were injected with a 1:5 split for leaf samples and 1:15 for stem samples. Gas chromatography was carried out with the oven temperature set to 50°C for 2 min, then raised by 40°C min^−1^ to 200°C and then held for 1 min and afterward raised by 3°C min^−1^ up to 320°C and then held for 15 min. The signals were integrated using the ChemStation Software (Agilent Technologies). Wax compounds were identified beforehand with gas chromatography linked with mass spectrometric detector (Agilent 5973 Network, Agilent Technologies). Quantification of wax amount was carried out by comparison of the peak areas from ionization detector to the internal standard. Limits of detection in leaves were as follows: alcohols: 0.0017 µg/cm^2^, alkanes: 0.0025 µg/cm^2^, fatty acids: 0.0016 µg/cm^2^ and aldehydes: 0.002 µg/cm^2^. Leaf and stem areas were determined as described (Haslam and Kunst 2013).

### Confocal microscopy

Aniline blue staining was used to visualize callose deposition after wounding. Whole *Arabidopsis* leaves were de-stained for 24 h in 1:3 (v/v) acetic acid:ethanol. Transparent leaves were washed in 150 mM K_2_HPO_4_ for 30 min and afterward incubated for 2 h in 150 mM K_2_HPO_4_ and 0.01% aniline blue. Samples were immersed in 50% glycerol for analysis. Tissues were examined with a Meta Confocal Laser Scanning Microscope (Carl Zeiss Mikroskopie, Jena, Germany) with a diode laser excitation at 405 nm.

### Statistical analysis

Data were statistically analyzed by a one-way ANOVA with Tukey’s post-hoc test using R studio v.1.1456.

## Supplementary Material

pcad137_Supp

## Data Availability

The data underlying this article are available in the article and in its online supplementary material.
