# Supplementary material for: Wounding Triggers Wax Biosynthesis in Arabidopsis Leaves in an Abscisic Acid–Dependent and Jasmonoyl-Isoleucine-Dependent Manner
Source: Plant Cell Physiol. 2023 Oct 31;65(6):928–38. doi: 10.1093/pcp/pcad137 (PMC11209552; doi:10.1093/pcp/pcad137)
Supplement: pcad137_Supp [file pcad137_supp.zip › pcp-2023-e-00147-File008.pdf]

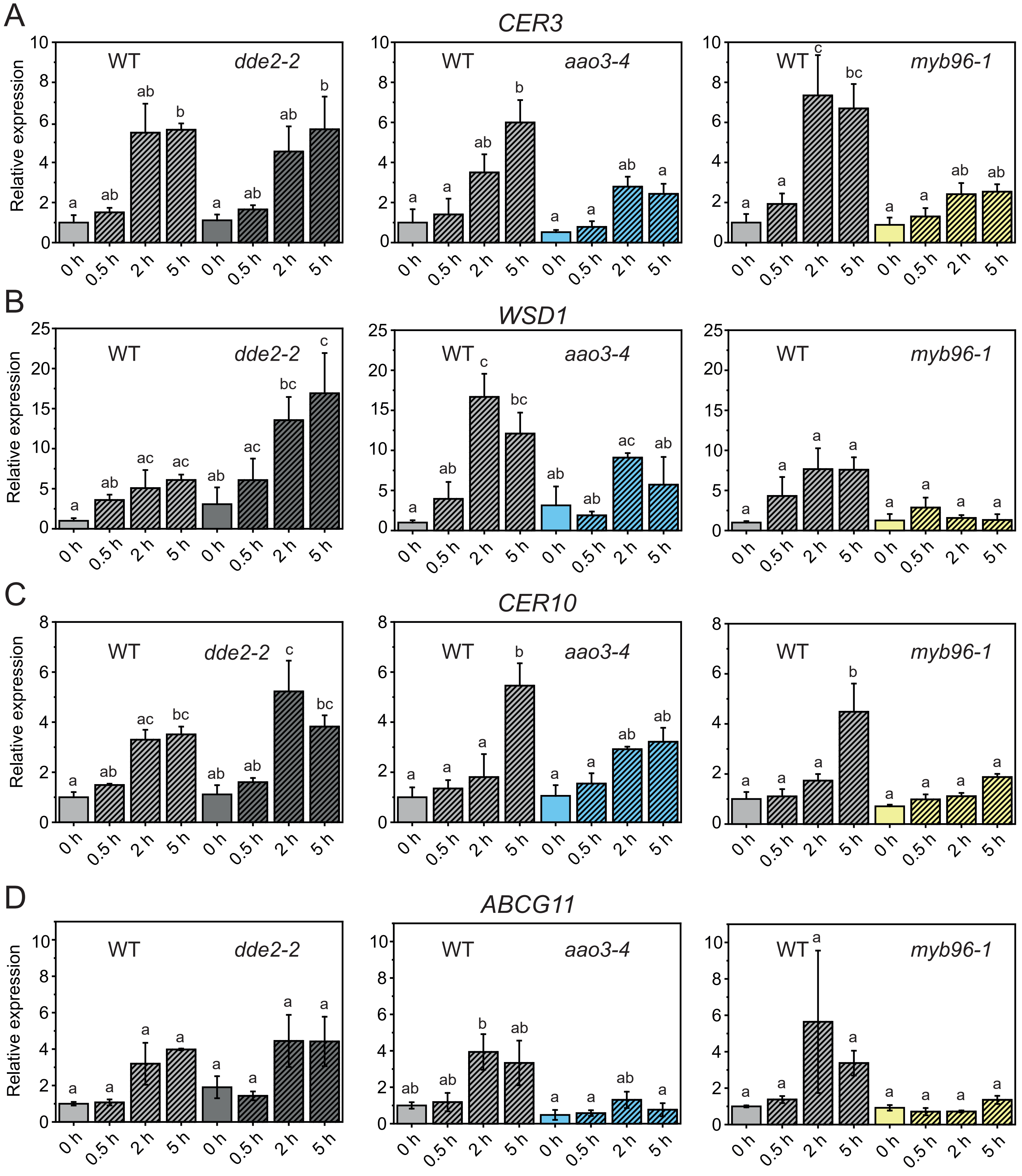

Figure S1. Expression of genes involved in wax biosynthesis is induced upon wounding. Transcript of chosen genes was analyzed by quantitative Real Time PCR (qRT-PCR) in leaves of WT, *dde2-2, aao3-4*, and *myb96-1* before (0 h), 0.5, 2, and 5 h post wounding. Relative expression of the following transcripts are shown: A, *CER3* involved in aldehyde/alkane formation; B, *WSD1* involved in wax ester synthesis; C, *CER10* involved in fatty acid elongation; D, *ABCG11* coding a wax transporter. Values represent means (±SE) of qRT- PCR analyses of plants of three independent experiments. Letters indicate statistical significance determined by ANOVA and Tukey‘s post-hoc test (P<0.05).

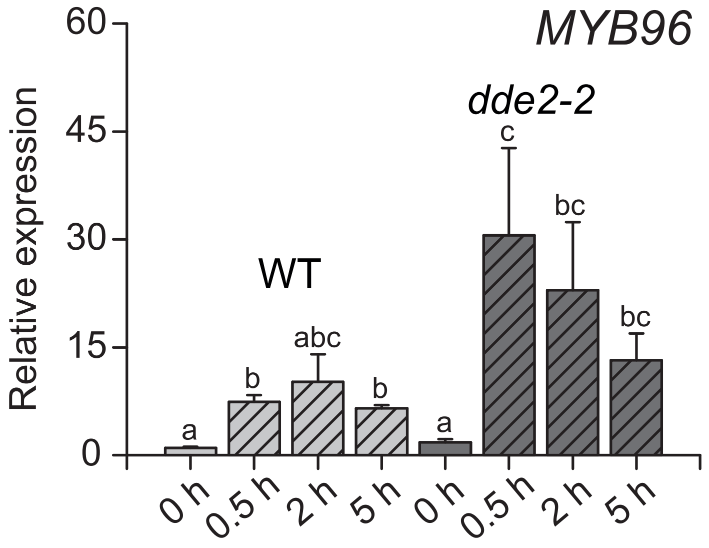

Figure S2. Expression of *MYB96* in WT and *dde2-2* before and after wounding.
Values are means (±SE) of quantitative Real Time PCR analyses of plants harvested from three independent wounding experiments. Letters indicate statistical significance determined by ANOVA and Tukey‘s post-hoc test (P<0.05).

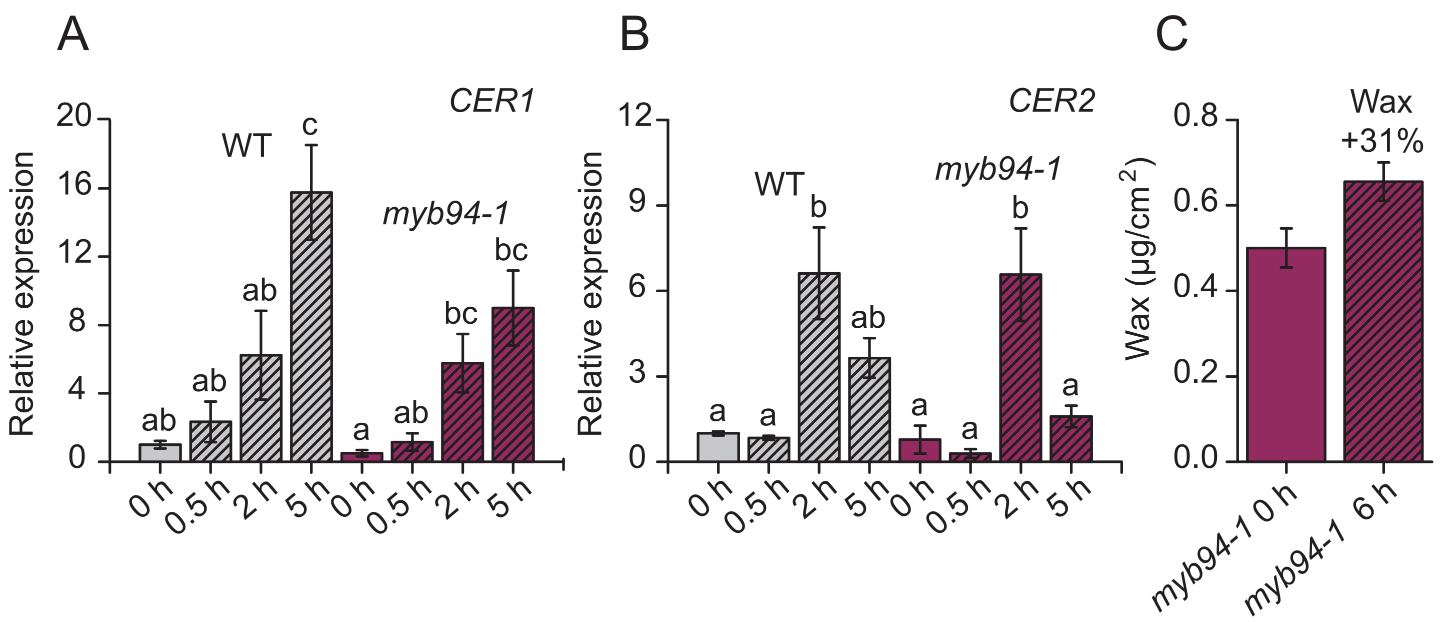

Figure S3. Response to wounding in *myb94-1*. A, expression of *CER1* and B, *CER2* in WT and *myb94-1* before (0 h) and 0.5, 2, 5 hours after wounding. Values represent means (±SE) of quantitative Real Time PCR analyses of plants harvested from three independent wounding experiments. Letters indicate statistical significance determined by ANOVA and Tukey‘s post-hoc test ($P<0.05$). C, wax content before and 6 h after wounding in *myb94-1*. Values represent means (±SD) of GC-FID analyses of 8 biological replicates.

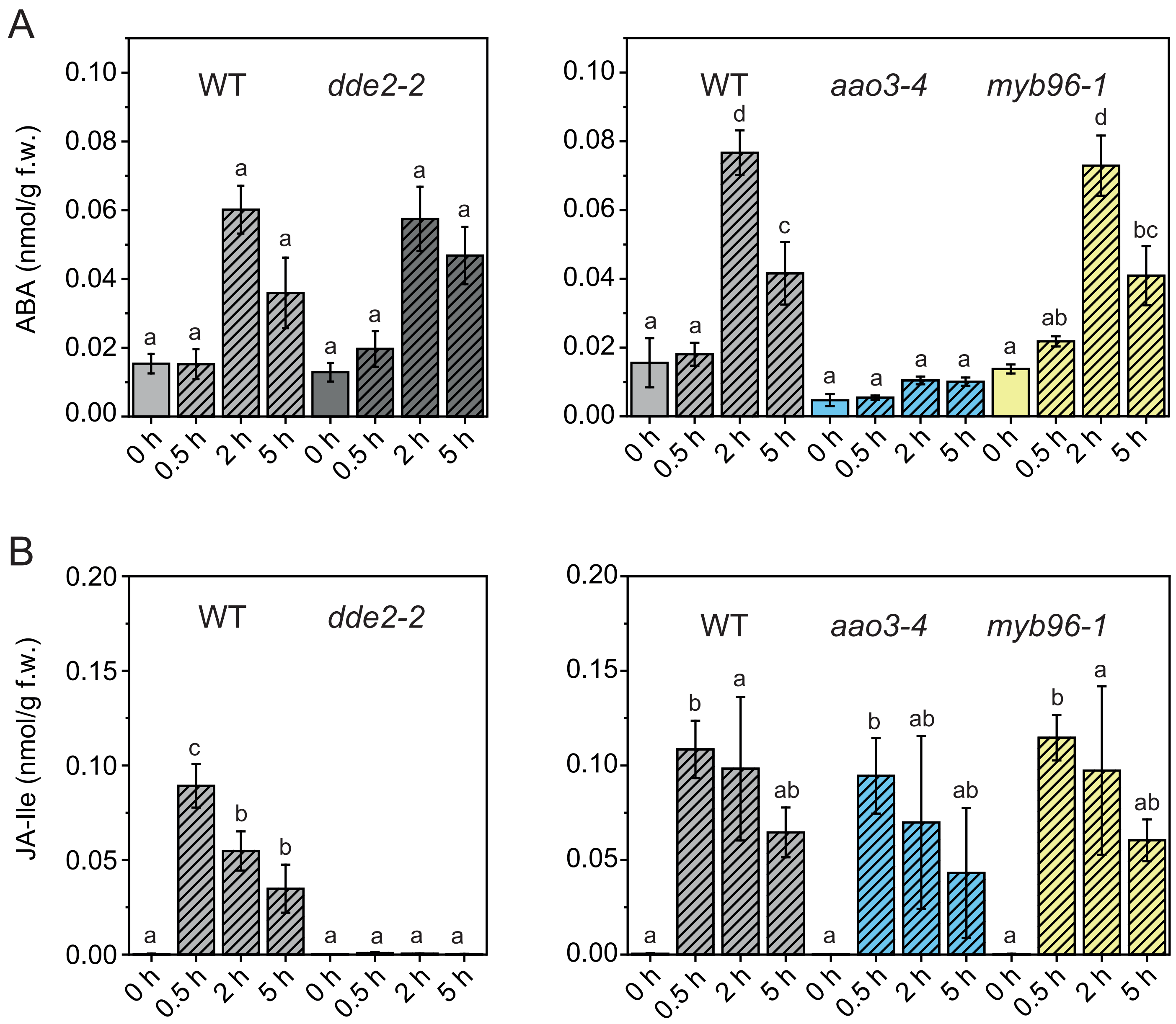

Figure S4. Analysis of JA-Ile and ABA amount in non-wounded and wounded leaves of WT, *dde2-2, aao3-4,* and *myb96-1* before (0 h) and 0.5, 2, 5 hours after wounding. A, ABA amount and B, JA-Ile amount before and after wounding. Values represent means (±SD) of LC-MS/MS analysis of plants harvested from three independent wounding experiments. Alphabetical letters indicate statistical significance determined by ANOVA and Tukey‘s post-hoc test (P<0,05).

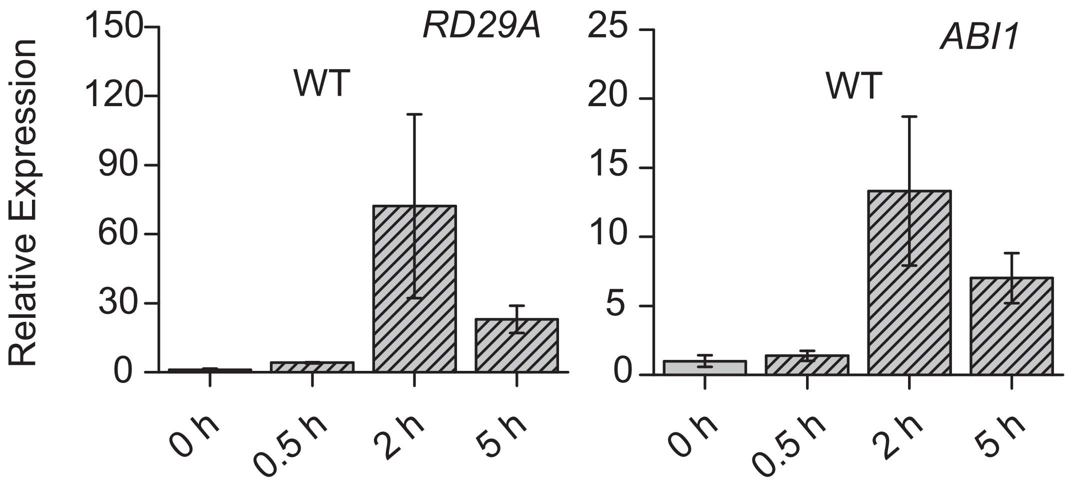

Figure S5. Expression of *RD29A* and *ABI1* involved in ABA signaling in abiotic stress response. Values represent means (±SD) of qRT-PCR analyses of plants harvested from three independent wounding experiments.
